# Analysis of ripening-related gene expression in papaya using an *Arabidopsis*-based microarray

**DOI:** 10.1186/1471-2229-12-242

**Published:** 2012-12-21

**Authors:** João Paulo Fabi, Graham B Seymour, Neil S Graham, Martin R Broadley, Sean T May, Franco Maria Lajolo, Beatriz Rosana Cordenunsi, João Roberto Oliveira do Nascimento

**Affiliations:** 1 University of São Paulo, Department of Food Science and Experimental Nutrition, FCF, São Paulo, Brazil; 2 Plant and Crop Sciences Division, School of Biosciences, University of Nottingham, Sutton Bonington Campus, Loughborough, Leics, LE12 5RD, UK; 3 University of São Paulo, – NAPAN – Food and Nutrition Research Center, São Paulo, Brazil

**Keywords:** Oligo-chip, Heterologous microarray, Papaya ripening, Quantitative gene expression, Whole genome shotgun, Transcript profiling

## Abstract

**Background:**

Papaya (*Carica papaya* L.) is a commercially important crop that produces climacteric fruits with a soft and sweet pulp that contain a wide range of health promoting phytochemicals. Despite its importance, little is known about transcriptional modifications during papaya fruit ripening and their control. In this study we report the analysis of ripe papaya transcriptome by using a cross-species (XSpecies) microarray technique based on the phylogenetic proximity between papaya and *Arabidopsis thaliana*.

**Results:**

Papaya transcriptome analyses resulted in the identification of 414 ripening-related genes with some having their expression validated by qPCR. The transcription profile was compared with that from ripening tomato and grape. There were many similarities between papaya and tomato especially with respect to the expression of genes encoding proteins involved in primary metabolism, regulation of transcription, biotic and abiotic stress and cell wall metabolism. XSpecies microarray data indicated that transcription factors (TFs) of the *MADS*-box, *NAC* and *AP2*/*ERF* gene families were involved in the control of papaya ripening and revealed that cell wall-related gene expression in papaya had similarities to the expression profiles seen in *Arabidopsis* during hypocotyl development.

**Conclusion:**

The cross-species array experiment identified a ripening-related set of genes in papaya allowing the comparison of transcription control between papaya and other fruit bearing taxa during the ripening process.

## Background

Papaya (*Carica papaya* L.) is an important crop cultivated in tropical and subtropical areas and the ripe fruit has a soft and sweet pulp with high amounts of pro-vitamin A and antioxidants [1]. Papaya is a typical climacteric fruit, with striking colour changes, a rapid rise in ethylene production, and substantial pulp softening; it also responds to exogenous ethylene and 1-MCP applications [2,3]. The physico-chemical changes during papaya ripening are dependent on the expression of specific genes, and the identification of ripening-related genes involved in the activation of biochemical steps relevant for fruit quality is of both scientific and commercial interest.

In order to understand the network of ripening genes in fleshy fruits, transcriptome studies are valuable tools. In the case of fruit such as tomato, microarrays have been used extensively [4,5]. However, for less well studied fruits, transcriptome analyses are based on ‘home-made’ microarrays, such as the μPEACH1.0 array [6], or classical transcript profiling by Differential Display-PCR or cDNA-AFLP [7-9]. With the development of high-throughput sequencing, several species have had their genome sequenced including the Hawaiian variety of papaya fruit [10]. Commercial oligo-chips are not currently available for these organisms and comprehensive RNA sequencing can still be costly often prohibiting routine experiments. However, a cross-species (XSpecies) microarray is an alternative approach that has been successfully used to study the transcriptomes of non-model organisms [11,12].

Papaya is a member of family Caricaceae within the Brassicales, the same order as the ‘model plant’ *Arabidopsis thaliana*, which has been the object of many microarray experiments based on commercially available oligo-chips. Because the two species are relatively closely related, the use of *Arabidopsis* arrays to hybridize RNA from papaya should provide information on the transcriptome changes during ripening in papaya fruits.

In the present study we report the use of RNA from unripe and ripe papaya to probe the Affymetrix *Arabidopsis* GeneChip ATH1-121501 to profile ripening–related gene expression in papaya. The expression pattern of a number of genes likely to be related to fruit quality was validated by quantitative real-time PCR, and the data from papaya cross-species microarray was compared to microarray data from tomato (a climacteric fruit) and grape (a non-climacteric fruit). A comparative biology approach was then used to compare the putative proteins from papaya and protein sequences from *Arabidopsis* and other fleshy fruits in order to obtain information on the differences between these organisms in respect to evolutionary role in fruit ripening. The expression of transcription factors was divergent amongst three species, and XSpecies data indicated transcription factors (TFs) that may be involved in the control of papaya ripening. Comparison of the expression patterns of ripening-related TFs and down-stream effectors such as cell wall genes between papaya, fleshy fruits and *Arabidopsis* indicated both common and unique features in these higher regulatory networks governing ripening.

## Results

### Transcriptome characteristics of papaya fruit ripening: Use of XSpecies microarray

A probe-masking strategy utilising hybridization of papaya genomic DNA was used to identify probes with low or non-specific hybridization. The number of probe-pairs retained for analysis decreased rapidly (Additional file 1) as the DNA hybridization threshold was increased. In comparison the number of probe-sets reduced at a slower rate, which was consistent with the results obtained with other species [11,12]. The number of differentially expressed genes (fold change >1.25, p<0.05) was calculated at each threshold and the mask value of 75 returned the highest number of differentially expressed putative genes (414 probe-sets) (Additional file 2). The hierarchical clustering of the log_2_ values of these probe-sets intensities resulted in the discrimination of eight main clusters (Additional file 3) with different expression patterns. Clusters II, III, IV, V and VI, with 208 probe-sets, were up-regulated, while 205 probe-sets from clusters I, VII and VIII were down-regulated during ripening. Clusters I, II and III was composed by genes with high levels of expression while clusters VI and VIII enclosed genes with the lowest levels of expression during papaya ripening. Clusters IV, V and VII enclosed genes with the highest differences in gene expression when log_2_ intensities were compared between unripe and ripe fruit.

**Figure 1:**
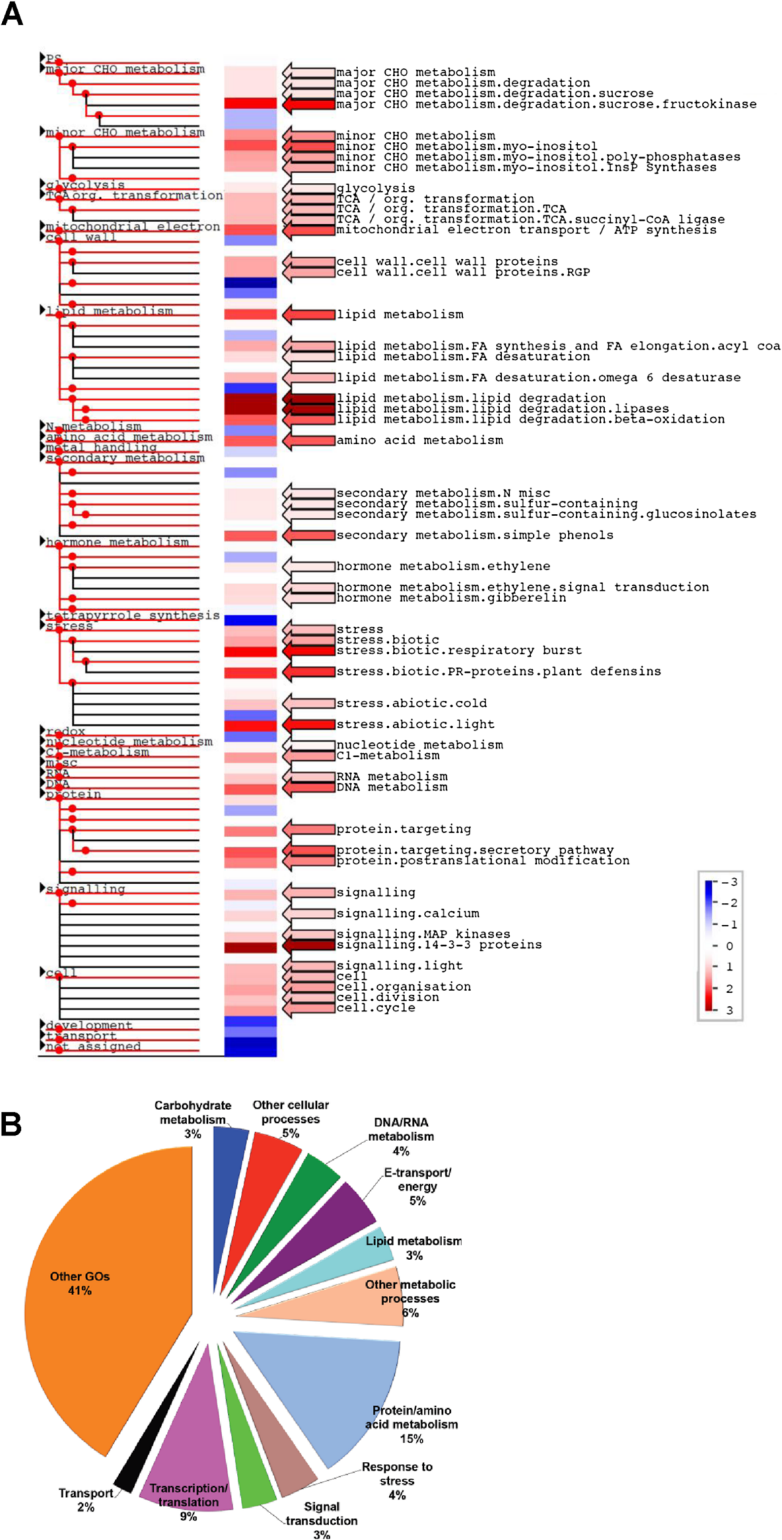
**The ripening papaya transcriptome was studied using the cross**-**species microarray technique and the Affymetrix ATH1-121501 GeneChip from *A. thaliana. *A**) Papaya normalized gene expression values were subjected to an analysis to identify overrepresented functional categories using PageMan (indicated by arrows and names). Gene expression data are presented as log_2_ fold changes in comparison with the unripe fruit. The data were subjected to a Wilcoxon test, and the results are displayed in false-color code. Red colored bins are significantly up-regulated, whereas blue colored bins are significantly down-regulated (p <0.05). All non-significant categories and some down-regulated bins are collapsed in the display. **B**) Pie chart showing the percentage of genes in ripe papaya that are over represented with respect to each of the GO biological processes.

Papaya probe-sets were separated into gene categories using the PageMan software and up-regulated probe-sets were organized according to the correspondent *Arabidopsis* Gene Ontology (Additional file 4) function (Figure 1). As was expected in a climacteric fruit, there were a wide range of genes up-regulated during fruit ripening, including those involved in primary metabolism (especially carbohydrate degradation) and energy transport and these findings are consistent with the high demand associated with ripening and the peak in CO_2_ production (as it was previously reported [3,8]) (Additional file 5). Genes encoding proteins involved in lipids, protein, and hormone metabolism, as well as cell signalling, signal transduction, and the response to biotic and abiotic stress were also up-regulated (Table 1).

**Table 1:** **Differentially expressed probe**-**sets identified in papaya fruit**

**TAIR annotation**	**Gene description** (**annotated by GO analysis**)	**Log**_**2**_**Fold**^**†**^	***P***-**value**
***Carbohydrate Metabolism***			
AT1G50390	Fructokinase-related (***FRUCT***)^††^	1.738	0.0358
AT3G08900	Reversibly glycosylatable polypeptide-related	0.759	0.0323
AT2G22240	Myo-inositol 1-phosphate synthase	0.748	0.0414
AT2G40220	Abscisic acid-insensitive 4 (*ABI4*) identical to AP2 domain transcription factor	0.361	0.0472
AT3G26380	Glycosyl hydrolase family 27 (alpha-galactosidase/melibiase) (***A***-***GAL***)	-0.342	0.0216
AT5G66280	GDP-D-mannose 4,6-dehydratase 1 (*GMD1*)	-0.386	0.0349
AT1G75940	Glycosyl hydrolase family 1 (beta-glucosidase)	-0.482	0.0402
AT2G06850	Xyloglucan endotransglycosylase (ext/*EXGT*-A1) (***XTH***)	-0.862	0.0498
AT4G28320	Glycosyl hydrolase family 5/cellulase ((1-4)-beta-mannan endohydrolase) (***CELL***)	-2.911	0.0228
***E***-***transport***/***Energy***			
AT5G51060	Respiratory burst oxidase protein C (NADPH oxidase)	1.809	0.0189
AT3G24200	Monooxygenase family	1.610	0.0234
AT2G29990	NADH dehydrogenase	1.108	0.0128
AT2G05180	Cytochrome P450	0.834	0.0275
AT5G08300	Succinyl-CoA-ligase alpha subunit	0.708	0.0194
AT2G36530	Enolase (2-phospho-D-glycerate hydroylase) (***ENOL***)	0.523	0.0400
AT3G56840	FAD dependent oxidoreductase	-0.593	0.0458
AT5G53460	Glutamate synthase	-0.744	0.0452
***Lipid Metabolism***			
AT3G62590	Lipase (class 3) (***LIP3***)	3.851	0.0367
AT1G53920	GDSL-motif lipase/hydrolase	1.710	0.0157
AT5G65110	Acyl-CoA oxidase (***ACYL***)	1.038	0.0070
AT1G30370	Lipase (class 3) similar to DEFECTIVE IN ANTHER DEHISCENCE1	0.972	0.0296
AT4G30950	Omega-6 fatty acid desaturase	0.725	0.0393
AT3G15850	Fatty acid desaturase similar to delta 9 acyl-lipid desaturase	0.344	0.0204
AT1G28590	Lipase (***LIP***)	-0.364	0.0373
***Protein***/***Amino Acid Metabolism***			
AT5G63860	UVB-resistance protein UVR8	1.625	0.0262
AT1G76700	DnaJ protein family (*DnaJ1*)	1.080	0.0340
AT1G14570	UBX domain-containing protein	0.674	0.0361
AT1G16030	Heat shock protein 70b (***HSP70***)	0.669	0.0157
AT4G17830	Aminoacylase similar to acetylornithine deacetylase	0.544	0.0053
AT4G23600	Aminotransferase similar to nicotianamine aminotransferase	0.534	0.0491
AT1G17720	Type 2A protein serine/threonine phosphatase	0.420	0.0292
AT4G00690	Ulp1 protease similar to SUMO-1/Smt3-specific isopeptidase 2	-0.427	0.0443
AT5G20890	Chaperonin	-0.438	0.0102
AT4G20850	Tripeptidyl-peptidase II	-0.474	0.0280
AT3G58640	Protein Kinase Family Protein	-0.599	0.0207
AT5G65940	3-hydroxyisobutyryl-coenzyme A hydrolase (CoA-thioester hydrolase)	-0.711	0.0376
AT5G22060	DnaJ protein family (*DnaJ3*)	-1.032	0.0416
***Response to Stress***/***Defense***			
AT2G43510	Trypsin inhibitor-related	1.401	0.0030
AT3G22840	Early light-induced protein	0.629	0.0440
AT3G17020	Expressed protein similar to *ER6* protein	0.679	0.0306
AT5G47100	Calcineurin B-like protein 9 (*CBL9*)	0.385	0.0326
AT3G16450	Jacalin lectin family similar to myrosinase-binding protein homolog	-0.402	0.0039
AT1G72950	Disease resistance protein	-0.565	0.0447
AT2G17310	F-box protein family	-1.211	0.0150
AT3G03670	Peroxidase	-1.831	0.0497
***Signal Transduction***			
AT2G30420	Myb family transcription factor	1.689	0.0456
AT2G24500	C2H2-type zinc finger protein -related	0.856	0.0103
AT3G61950	bHLH protein family	0.589	0.0216
AT2G06020	Myb family transcription factor	-1.482	0.0367
***Transcription***/***Translation***			
AT3G61830	Auxin response factor-related protein 18 (***ARF18***)	1.004	0.0247
AT3G55620	Eukaryotic translation initiation factor 6 (*EIF*-*6*)	0.743	0.0050
AT1G15360	*AP2*/*ERF transcription factor 2* (***ERF2***)	0.596	0.0407
AT4G11160	Translation initiation factor IF-2 (Basic helix-loop-helix family protein)	0.579	0.0272
AT5G60910	*MADS*-box protein (AGL8)	0.462	0.0135
AT3G24050	*GATA* transcription factor 1	0.364	0.0307
AT5G06950	bZIP transcription factor	-0.327	0.0262
AT3G05860	*MADS*-box protein	-0.357	0.0030
AT1G05180	Auxin-resistance protein 1 (***AXR1***)	-0.472	0.0230
AT1G46768	*AP2* domain protein *RAP2*.*1* (***RAP2***.***1***)	-0.730	0.0492
AT1G58110	bZIP family transcription factor	-1.311	0.0243
***Transport***			
AT4G18290	Inward rectifying potassium channel (*KAT2*)	1.868	0.0166
AT5G59030	Copper transport protein	1.015	0.0171
AT3G05165	Sugar transporter	-0.711	0.0453
AT3G12390	Nascent polypeptide associated complex alpha chain	-0.801	0.0182
AT1G77990	Sulfate transporter -related	-1.269	0.0347
***Other GOs***			
AT5G06650	Zinc finger-related protein (**GO**: **trichome differentiation**)	3.432	0.0429
AT5G16050	*14*-*3*-*3* protein GF14 upsilon (grf5) (**GO**: **unknown**)	1.466	0.0369
AT5G65430	*14*-*3*-*3* protein GF14 kappa (grf8) (**GO**: **unknown**)	1.450	0.0370
AT5G01190	Laccase (diphenol oxidase) (**GO**: **lignin catabolic process**)	1.024	0.0060
AT3G08900	Reversibly Glycosylated Polypeptide 3) (**GO**: **cellulose biosynthetic process**)	0.759	0.0323
AT3G12290	Tetrahydrofolate dehydrogenase (**GO**: **folic acid biosynthesis**)	0.778	0.0222
AT1G70140	Formin homology 2 (*FH2*) domain-containing protein (**GO**: **cell tip growth**)	0.760	0.0241
AT5G66170	Senescence-associated protein (**GO**: **aging**)	0.742	0.0069
AT5G07200	Gibberellin 20-oxidase (**GO**: **gibberellin biosynthetic process**)	0.578	0.0156
AT1G73690	Cell division protein kinase (**GO**: **regulation of cell cycle**)	0.506	0.0341
AT5G67160	Hydroxycinnamoyl/benzoyltransferase-related protein (**GO**: **unknown**)	0.504	0.0467
AT5G48450	Pectinesterase (pectin methylesterase) (***PME1***) (**GO**: **cell tip growth**)	0.501	0.0479
AT1G04130	Tetratricopeptide Repeat (TPR)-containing Protein (**GO**: **unknown**)	-0.347	0.0496
AT1G67700	Auxin-regulated protein (***ARP***) (**GO**: **unknown**)	-0.396	0.0364
AT4G12420	Pectinesterase (pectin methylesterase) (***PME2***) (**GO**: **cell tip growth**)	-0.421	0.0226
AT5G45360	F-box protein similar to SKP1 interacting partner 2 (SKIP2) (**GO**: **unknown**)	-0.448	0.0406
AT2G39700	Expansin putative (***EXP***) (**GO**: **cell wall organization and biogenisis**)	-0.474	0.0128
AT2G39750	Early-responsive to dehydration stress protein (***ERD3***) (**GO**: **unknown**)	-0.642	0.0294
AT4G04340	Early-responsive to dehydration stress protein (***ERD4***) (**GO**: **unknown**)	-0.749	0.0256
AT2G38700	Mevalonate diphosphate decarboxylase (***MEV***) (**GO**: **isoprenoid biosynthesis**)	-0.793	0.0224
AT4G24780	Pectate lyase family 1 (***PL***) (**GO**: **cell wall organization and biogenesis**)	-1.228	0.0408

^†^Log2 of average signal value ripe divided by average signal value unripe.

^††^Names of the genes analyzed by qPCR-Real Time.

### Comparative genomics of fruit ripening in papaya vs. Tomato and grape

In order to visualize the probable cell functions altered in papaya and to compare them to those of another climacteric fruit (tomato) and a non-climacteric fruit (grape), the bins of differentially expressed probe-sets for papaya (fold change >1.25, p<0.10; Additional file 6), tomato and grape (fold change >2.0, authors’ statistical cut-off) were run using the MapMan software [13]. The aim was identify sets of expression profiles that might be conserved between or unique to climacteric and non-climacteric fruit. Data chosen for analysis focused on the main changes associated with the ripening process, such as the comparison between red and mature green tomato and post-véraison and pre-vérasion grape.

**Figure 2:**
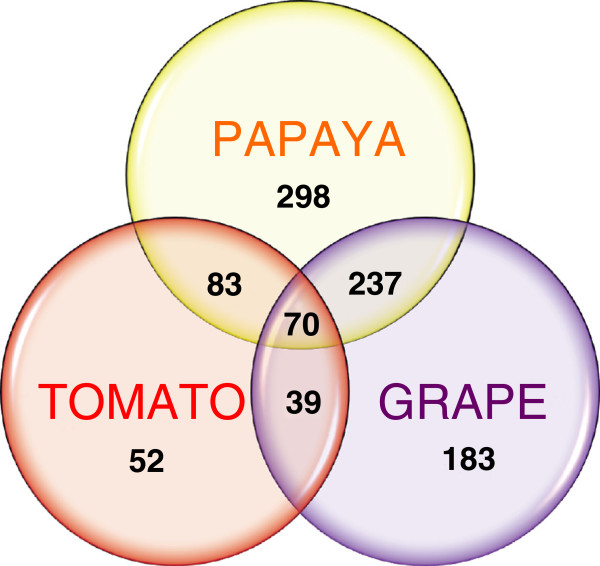
**Venn diagram representing the distribution of papaya**, **tomato and grape up**-**regulated genes obtained from microarray data. **Numbers within circles show the number of exclusive up-regulated genes and numbers within intersections show common up-regulated genes. A threshold value of 0.3 was used to construct the diagram based on log_2_ fold values. It was possible to group 962 genes from 1091 genes from papaya (> 1.25 fold), 1144 genes from tomato (> 2.00 fold) and 1150 genes from grape (> 2.00 fold). Tomato microarray data was downloaded from http://ted.bti.cornell.edu (Ozaki et al., 2010); grape microarray data was downloaded from http://biomedcentral.com (Pilati et al., 2007).

Figure 2 shows a schematic Venn diagram of up-regulated probe-sets from papaya XSpecies, tomato and grape microarrays obtained from MapMan analysis (Additional file 7). Despite differences between climacteric and non-climacteric ripening, the proportion of up-regulated genes varied amongst species, with a similar number of over-expressed genes between ripe papaya and grapes.

**Figure 3:**
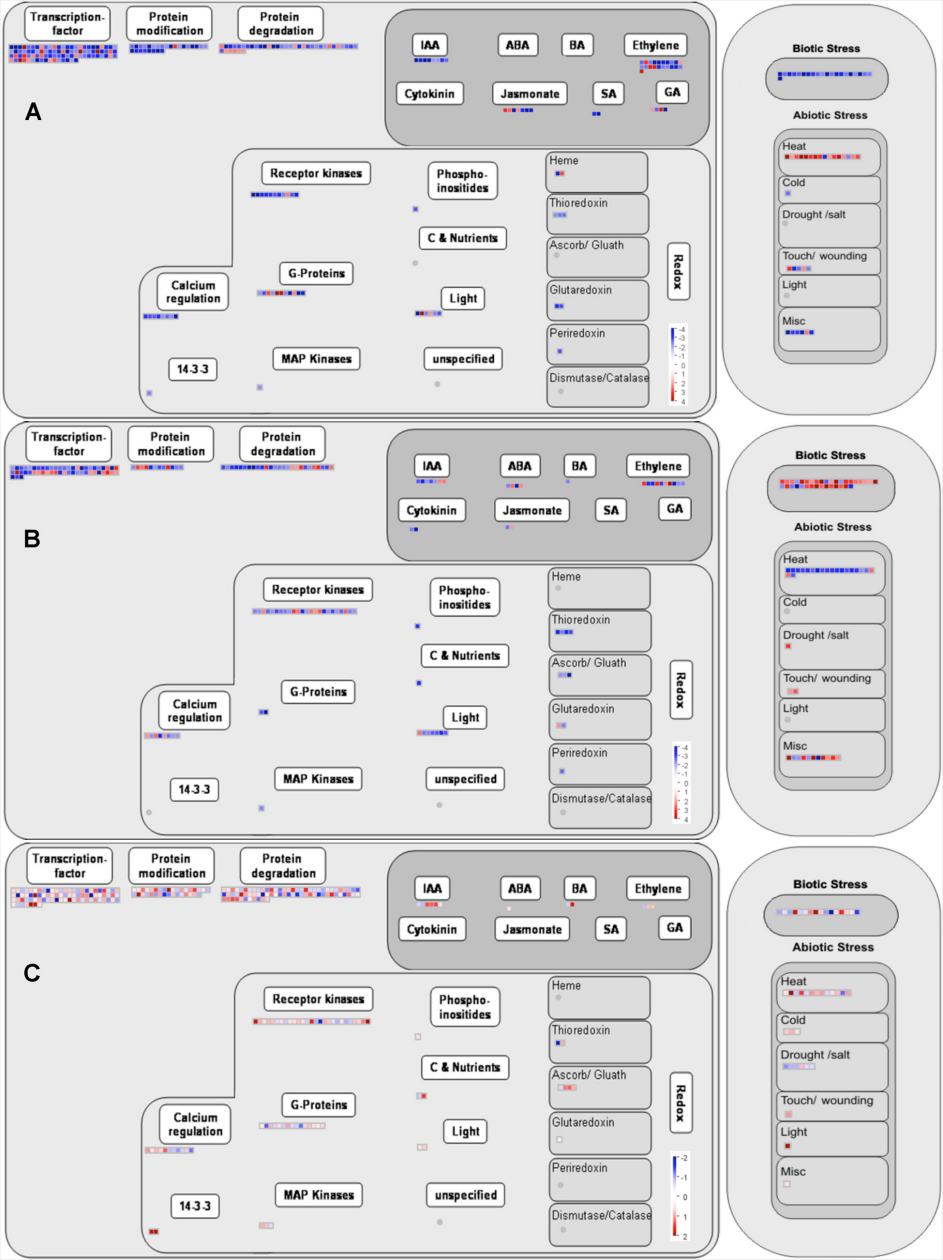
**Overview of ripening regulation and cellular responses to ripening from tomato**, **grape and papaya. **Transcripts from tomato (**A**), grape (**B**) and papaya (**C**) fruits were analysed using the MapMan software uploaded with microarrays results. Blue and red represent a decrease and an increase of expression respectively, relative to unripe fruits. It is visible 244 transcripts (from 1144) for tomato (> 2.00 fold), 171 transcripts (from 1150) for grape (> 2.00 fold) and 276 transcripts (from 1091) for papaya (> 1.25 fold). Tomato microarray data was downloaded from http://ted.bti.cornell.edu (Ozaki et al., 2010); grape microarray data was downloaded from http://biomedcentral.com (Pilati et al., 2007).

A schematic overview of ripening regulation and some of its cellular responses (Figure 3) showed a high proportion of up-regulated transcription factors (TFs) in papaya (61%) (Additional file 8). When probe-sets from papaya experiments were analysed in terms of GO molecular function (Additional file 4), 103 probe-sets (25%) accounted for genes responsible for nucleotides and nucleic acids binding, translation initiation factors and ribosomes constituents. Table 2 shows the main up-regulated TFs in the three species regarding the three different ripening experiments.

**Table 2:** **Differentially expressed transcription factors from papaya**, **tomato and grape**

**Gene description**	**TAIR annotation**	**Log**_**2**_**Fold**^†^			**Probe Set ID**
		**Papaya**	**Tomato**^††^	**Grape**^†††^	
*AP2* domain protein *RAP2*.*1* (***RAP2***.***1***)	AT1G46768	−0.73			245807_at
*AP2 domain*-*containing protein*	AT5G52020	-	4.15	-	lesaffx.63544.1.s1_at
*AP2 domain*-*containing transcription factor*	AT4G39780	0.54	-	-	252859_at
*AP2*/*ERF transcription factor*	AT2G47520	-	2.18	-	les.4102.1.s1_at
*AP2*/*ERF transcription factor 2* (***ERF2***)	AT1G15360	0.60	-	-	262595_at
*ATHB23* (*A*. *thaliana HOMEOBOX PROTEIN 23*)	AT1G26960	0.41	-	-	263690_at
*Auxin response factor*-*related protein 18* (***ARF18***)	AT3G61830	1.00	-	-	251289_at
*Basic helix*-*loop*-*helix* (*bHLH*) *family protein*	AT2G34820	0.55	-	-	267426_at
*Basic helix*-*loop*-*helix* (*bHLH*) *family protein*	AT3G61950	0.59	-	-	251299_at
*Basic helix*-*loop*-*helix* (*bHLH*) *family protein*	AT4G09180	-	2.20	-	lesaffx.19952.1.s1_at
*BLH7* (*BELL1*-*LIKE HOMEODOMAIN 7*)	AT2G16400	1.76	-	-	263557_at
*C2H2*-*type zinc finger protein* -*related*	AT2G24500	0.86	-	-	265662_at
*Embryo*-*abundant protein*-*related*	AT2G41380	-	-	3.26	1620276_at
*Eukaryotic translation initiation factor 6* (*EIF*-*6*)	AT3G55620	0.74	-	-	251776_at
*GATA transcription factor 1*	AT3G24050	0.36	-	-	256916_at
*KNAT3* (*KNOTTED1*-*LIKE HOMEOBOX GENE 3*)	AT5G25220	-	2.45	-	lesaffx.67017.1.s1_at
*MADS*-*box protein* - *Floral homeotic protein PISTILLATA* (*PI*)	NM*	-	-	3.02	1614123_at
*MADS*-*box protein* (*AGL20*)	AT2G45660	-	-	2.53	1612908_at
*MADS*-*box protein* (*AGL8*)	AT5G60910	0.46	-	-	247553_at
*MADS*-*box protein* (*AGL8*) - *TDR4 transcription factor*	AT5G60910	-	1.38	-	les.4461.1.S1_s_at
*MADS*-*box transcription factor* (*AGL2* - *MADS*-*RIN*)	AT5G15800	-	3.97	-	les.4450.1.s1_at
*MYB family transcription factor*	AT2G30420	1.69	-	-	267495_at
*MYB family transcription factor*	AT5G08520	0.83	-	-	250524_at
*MYB family transcription factor*	AT5G45420	0.80	-	-	248954_at
*MYB10* (*myb domain protein 10*)	AT3G12820	0.72	-	-	257689_at
*MYB111* (*myb domain protein 111*)	AT3G46130	-	2.58	-	les.4982.1.s1_at
*MYB19* (*myb domain protein 19*)	AT5G52260	0.39	-	-	248343_at
*MYB21* (*myb domain protein 21*)	AT3G27810	-	5.75	-	lesaffx.70738.1.a1_at
*MYB43* (*myb domain protein 43*)	AT5G16600	-	2.66	-	lesaffx.64717.1.a1_at
*MYB73* (*myb domain protein 73*)	AT4G37260	0.34	-	-	246253_at
*MYB75* (*myb domain protein 75*) - *PRODUCTION OF ANTHOCYANIN PIGMENT 1*	NM	-	-	3.74	1620959_s_at
*MYB95* (*myb domain protein 95*)	AT1G74430	0.46	-	-	260237_at
*MYR1* (*MYB*-*RELATED PROTEIN 1*)	AT3G04030	0.43	-	-	258807_at
*NAC domain protein* (*NAC13*)	AT1G32870	0.79	-	-	261192_at
*NAC domain protein* (*NAC4*)	NM	-	-	2.05	1610466_at
*NAC domain protein* (*NAC42*)	AT2G43000	-	1.30	-	lesAffx.66359.1.S1_at
*NAC domain protein* (*NAC47*)	AT3G04070	-	-	2.20	1607620_at
*Zinc finger* (*C2H2 type*) *family protein*	AT1G68130	-	-	2.07	1613842_at
*Zinc finger* (*C2H2 type*) *family protein*	AT5G66730	1.07	-	-	247054_at

^†^ Log_2_ of average signal value ripe divided by average signal value unripe;

^††^ Microarray data downloaded from http://ted.bti.cornell.edu (Ozaki et al., 2010);

^†††^ Microarray data downloaded from http://biomedcentral.com (Pilati et al., 2007);

* NM: no match with TAIR sequences.

The cellular response to ripening in these three fruits was also different, especially to biotic and abiotic stress and redox regulation (Figure 3). While tomato responses were the increase of genes related to abiotic stress and decrease of those related to biotic stress, grape responded in an opposite way (Table 3). Nonetheless, papaya responded to ripening in a similar way to tomato, with abiotic genes being expressed in the same time course than tomato ones.

**Table 3:** **Differentially expressed probe**-**sets related to cellular response to ripening**

**Gene Description**	**TAIR annotation**	**Log**_**2**_**Fold**^†^			** Probe set ID**
		**Papaya**	**Tomato**^††^	**Grape**^†††^	
***Biotic stress***					
*Acidic endochitinase* (*CHIB1*)	AT5G24090		-2.98		les.435.1.s1_at
*Acidic endochitinase* (*CHIB1*)	AT5G24090			2.84	1612050_at
*ATEP3* (*Arabidopsis thaliana chitinase class IV*)	AT3G54420		-5.52		lesaffx.69659.1.s1_at
*ATLP*-*1* (*Arabidopsis thaumatin*-*like protein 1*)	AT1G18250		-2.57		lesaffx.66226.2.s1_at
*Chitinase*, *putative*	AT2G43580	-0.50			260561_at
*Defensin*-*like* (*DEFL*) *family protein*	AT5G43510	-1.08			249157_at
*Disease resistance family protein* / *LRR family protein*	AT3G23010	0.37			257764_at
*Disease resistance protein* (*CC*-*NBS*-*LRR class*)	AT1G12290	1.22			259534_at
*Disease resistance protein* (*TIR*-*NBS*-*LRR class*)	AT5G44510	0.47			249058_at
*Glyco*_*hydro*_*19 chitinase*	AT2G34690			2.76	1620505_at
*LCR70* (*Low*-*molecular*-*weight cysteine*-*rich 70*)	AT2G02120	0.86			266141_at
*Pathogenesis*-*related protein*	AT4G33720		-2.22		les.4693.1.s1_at
*Pathogenesis*-*related thaumatin family protei*	AT1G20030	-0.71			261248_at
*Polygalacturonase inhibiting protein 1* (*PGIP1*)	AT3G23170			2.07	1613339_at
*RHD2* (*ROOT HAIR DEFECTIVE 2*)	AT5G51060		-2.35		les.335.1.s1_at
*RHD2* (*ROOT HAIR DEFECTIVE 2*)	AT5G51060	1.80			248486_at
*Thaumatin*-*like protein*	AT1G77700			2.76	1607225_at
*Thaumatin*-*like protein*	AT1G75030			2.31	1616617_at
***Abiotic stress***					
*15*.*7 kDa class I*-*related small heat shock protein*-*like*	AT5G37670		2.03		lesaffx.70264.1.s1_at
*18*.*1 kDa class I heat shock protein* (*HSP18*.*1*)	AT3G23170			-2.46	1612385_at
*DNAJ heat shock family protein*	AT2G22360	-0.49			264002_at
*DNAJ heat shock N*-*terminal domain*-*containing protein*	AT1G56300		2.15		lesaffx.68054.1.s1_at
*DNAJ heat shock N*-*terminal domain*-*containing protein*	AT2G42750			-2.92	1609580_at
*DNAJ heat shock N*-*terminal domain*-*containing protein*	AT3G58020	-0.56			251618_at
*DNAJ heat shock N*-*terminal domain*-*containing protein*	AT1G76700	1.08			259876_at
*HSC70*-*1* (*heat shock cognate 70 kDa protein 1*)	AT1G74310			-2.70	1621357_s_at
*HSP101* (*heat shock protein 101*)	AT1G74310			-2.70	1615503_at
*HSP18*.*2* (*HEAT SHOCK PROTEIN 18*.*2*)	AT5G59720	0.66			247691_at
*HSP20*-*like chaperone*	AT3G22530	1.68			256934_at
*HSP70* (*heat shock 70 kDa protein*)	AT2G32120	0.75			265675_at
*HSP70* (*heat shock protein 70*)	AT3G12580		2.00		lesaffx.10807.1.s1_at
*HSP81*-*1* (*heat shock protein 81*-*1*)	AT5G52640		2.90		les.3134.1.s1_at
***Redox*** (***ascorbate and glutathione***)					
*Glutathione peroxidase*	NM*			-4.84	1614945_a_at
*L*-*ascorbate peroxidase*	AT4G09010			-2.12	1618209_at
*L*-*galactose dehydrogenase* (*L*-*GalDH*)	AT4G33670	0.66			253307_at
*Monodehydroascorbate reductase*	AT3G09940	0.87			258941_at

^†^ Log_2_ of average signal value ripe divided by average signal value unripe.

^††^ Microarray data downloaded from http://ted.bti.cornell.edu (Ozaki et al., 2010).

^†††^ Microarray data downloaded from http://biomedcentral.com (Pilati et al., 2007).

* NM: no match with TAIR sequences.

### Validation of gene expression and comparative biology between papaya and fleshy fruit organs

**Figure 4:**
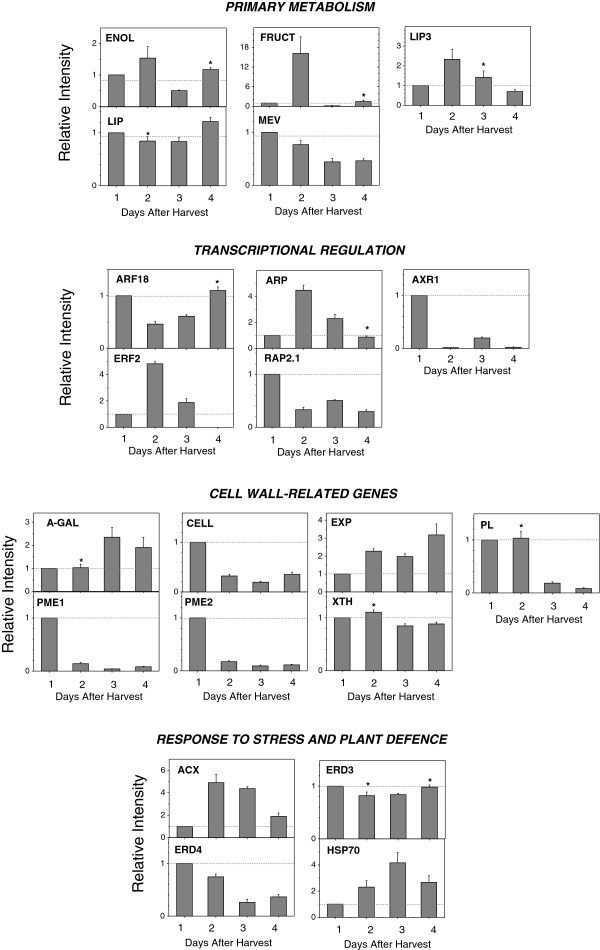
**QPCR validation of papaya ripening**-**related gene expression. **Real-time PCR was used to analyse the expression patterns of putative genes during papaya ripening with days after harvest. Column height indicates relative mRNA abundance; expression values in unripe fruit at the first day after harvest were set to 1. All data were normalised to the actin and 18S expression levels. Error bars on each column indicate SDs from four technical replicates. Asterisks represent samples that were not significantly different compared to the first day after harvest using one-way ANOVA and the Tukey test (α<0.05, *n*=4). Different boxes represent each issue pointed in the **Discussion** (*Primary metabolism*, *Transcriptional regulation*, *Cell wall*-*related genes*, *Response to stress and plant defence*).

In order to validate the XSpecies experiment, the expression of 21 probe-sets that were satisfactory aligned to *Arabidopsis* genes were analysed by qPCR throughout papaya ripening. Papaya genes were named according to the *Arabidopsis* functions and grouped together, as shown in Figure 4. Quantitative analysis revealed *A*-*GAL*, *EXP*, *FRUCT*, *LIP3*, *ACX*, *ARP*, *ENOL*, *HSP70* and *ERF2* were induced during ripening, while *ARF18*, *CELL*, *MEV*, *LIP*, *PME1*, *PME2*, *PL*, *RAP2*, *XET*, *ERD3*, *ERD4* and *AXR* were decreased during ripening. Contrasting results between qPCR and XSpecies experiments were observed for three genes (*ARF18*, *EXP* and *PME1*).

**Figure 5:**
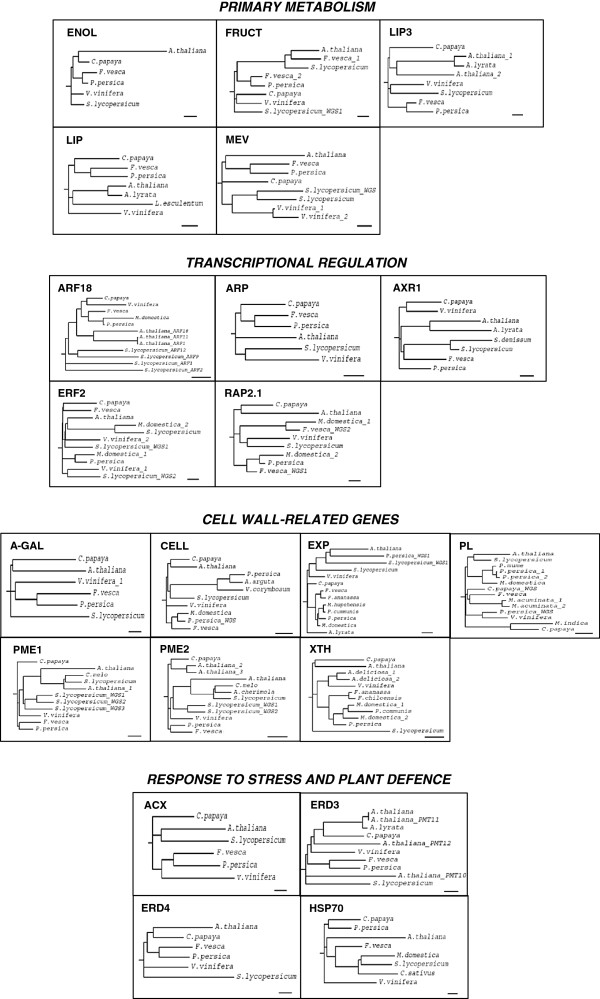
**Unrooted phylograms encompassing putative proteins from papaya, *A. thaliana *and several fleshy fruit organs. **Phylogenetic trees were calculated using Neighbor Joining method based on the ClustalW alignment of deduced amino acids sequences. Branch length values are based on the scale bar meaning 0.1 residue substitutions per site. Different boxes represent each issue pointed in the **Discussion** (*Primary metabolism*, *Transcriptional regulation*, *Cell wall*-*related genes*, *Response to stress and plant defence*).

Comparative analysis of the translated amino acids sequences for all genes analysed from qPCR experiments indicated some papaya putative proteins were more similar to those from species with fleshy fruits than to the *Arabidopsis* ones (Figure 5). In general, proteins from primary and secondary metabolism (*FRUCT*, *LIP*, *MEV*) and transcription factors (*ERF2*, *ARF18*, *AXR1*, *ARP*) appeared to be phylogenetically closer to those from species with fleshy fruits, whereas abiotic stress (*ERD3*, *ERD4*) and cell wall metabolism (*AGAL*, *PL*, *PME1*, *PME2*, *CELL*, *XTH*) proteins are more closely related to those from *Arabidopsis*. Contrasting results were observed for proteins with high degree of conservation, such as *ENOL*, *RAP2*, *HSP*, *ACX* and *PL*, where homology was determined primarily by non-conserved regions and less by conserved domains. Because cell wall related genes shared greater homology with *Arabidopsis*, a comparison of gene expression in ripe papayas was conducted using a published *Arabidopsis* microarray study of gene expression in 5 or 11-day-old hypocotyls (ratio 11-day/5-day) [14]. These data (Table 4) also indicate that this set of genes had comparable gene expression levels and might act on cell wall polymers in a similar way.

**Table 4:** **Cell wall**-**related genes from ripe papaya and *A***. ***thaliana *hypocotyl**

**Gene Description**	**TAIR annotation**	**Log**_**2**_**Fold**^†^		**Probe set ID**
		**Papaya**	***A***. ***thaliana***^††^	
*Pectate lyase* (*PL*)	AT1G09910	−1.01	−0.06	264658_at
*Expansin A15* (*EXPA15*)	AT2G03090	−0.42	−0.01	266770_at
*Xyloglucan endotransglycosylase* (*ext*/*EXGT*-*A1*) (*XTH*)	AT2G06850	−0.86	−0.65	266215_at
*UDP*-*glucuronate 4*-*epimerase* (*GAE6*)	AT3G23820	−1.55	−0.52	256865_at
*Cellulose synthase*-*like 12* (*CSL12*)	AT4G07960	0.34	−0.07	255175_at
*Pectinesterase* (*pectin methyl esterase*) (*PME2*)	AT4G12420	−0.42	−0.27	254815_at
*Pectate lyase family 1* (*PL*)	AT4G24780	−1.23	0.67	254119_at
*Cellulose synthase*-*like 9* (*CSL9*)	AT5G03760	−0.6	−0.64	250892_at
*COBRA*-*like gene 4* (*COBL4*)	AT5G15630	0.66	−0.47	246512_at
*Pectinesterase* (*pectin methylesterase*) (*PME1*)	AT5G48450	0.5 (−3.42)^*^	−0.36	248704_at
*Arabinogalactan protein 22* (*AGP22*)	AT5G53250	1.13	0.96	248252_at
*GDP*-*mannose 4*,*6*-*dehydratase 1* (*GMD1*)	AT5G66280	−0.42	−0.24	247094_at

^†^ Log_2_ of average signal value ripe vs. unripe for papaya and log_2_ of average signal value 11-day-old hypocotyls vs. 5 days for *A*. *thaliana*.

^††^ Microarray data downloaded from http://www.biomedcentral.com/content/supplementary/1471-2164-10-505-s1.pdf (Jamet et al. 2009).

^†††^ Number represented by qPCR analysis.

## Discussion

In this study we investigated the transcriptome of ripening papaya [7,8] using fruits at two contrasting physiological stages and the Affymetrix ATH1-121501 GeneChip for *Arabidopsis* in an XSpecies microarray [11]. The number of differentially expressed probe-sets was consistent with the results obtained with other species [11,12] but was fewer than those from regular microarray experiments, especially with regarding comparative analyses between unripe and ripe fruits [15,16]. Although XSpecies results have less power than a regular microarray experiment and technologies such as RNAseq performed by Next Generation Sequencing (NGS), the technique was able to identify important genes related to metabolic processes involved in papaya ripening. Surprisingly, despite papaya and *Arabidopsis* being members of the order Brassicales, only cell wall-related genes shared a greater homology among the genes studied. This may reflect shared metabolic pathways which differ from other fleshy fruit. Fleshy fruits showed greater homology between transcription factors which might indicate shared transcriptional regulatory networks.

### Deduced roles of ripening-related genes in papaya

#### Primary metabolism

The high energy demand at climacteric ripening was evidenced by the up-regulation of genes related to Krebs and TCA cycles (AT5G08300, AT2G29990, AT3G24200 and AT5G51060), cytochromes (AT2G05180, AT5G06900, AT1G01280), and hexose metabolism (AT2G36530 and AT1G50390). Similarly, genes involved in lipid metabolism, were also affected. These included cell membrane lipases (AT3G62590, AT1G53920 and AT1G28590), and up-regulation of genes responsible for the synthesis of unsaturated fatty acids (AT4G30950 and AT3G15850). Down-regulation of the *MEV* gene (AT2G38700) suggests the main precursors for volatiles production and carotenoid biosynthesis could be produced by the MEP pathway inside the plastids during papaya ripening [17]. Fabi et al. [8] identified another transcript related to *MEV* gene, suggesting mevalonate accumulation prior ripening.

#### Transcriptional regulation

When the probe-sets were analysed in terms of GO molecular function (Additional file 4), a high number of genes responsible for nucleotide and nucleic acid binding, translation initiation and ribosomes constituents were observed, indicating control of ripening at both the level of transcription and translation. Five members of the *MYB* family, which is important for ripening since *MYB* genes are known to regulate secondary metabolism and colour accumulation [18], were differentially expressed during ripening. Since colour of papaya pulp is correlated with accumulation of carotenoids [3], papaya *MYB* TFs might show distinct role in other metabolism during ripening.

**Figure 6:**
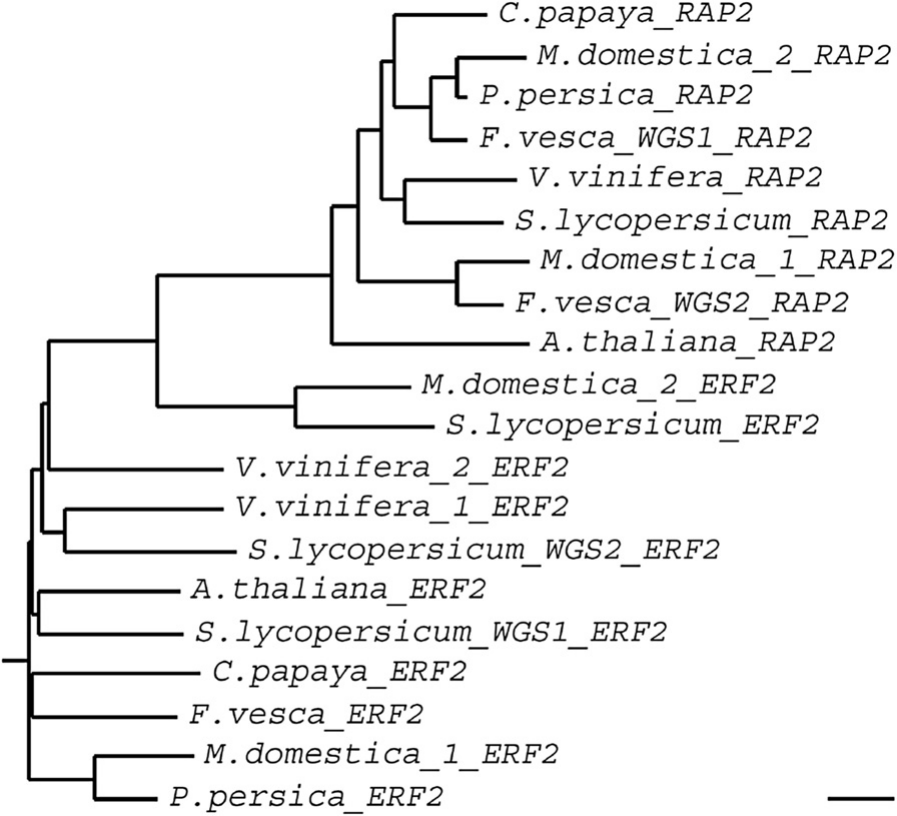
**Unrooted phylogram encompassing putative RAP2 and ERF2 proteins from papaya, *A. thaliana *and various other fleshy fruits. **The phylogenetic tree was calculated using Neighbor Joining method based on the ClustalW alignment of deduced amino acids sequences. Branch length values are based on the scale bar meaning 0.1 residue substitutions per site. TFs protein sequences from *RAP2* are: *C*. *papaya* (ABIM01006309), *A*. *thaliana* (ABD57516), *M*. *domestica*_*1* (ADE41138), *M*. *domestica*_*2* (ADE41135), *V*. *vinifera* (XP_002284933), *S*. *lycopersicum* (AEKE02013217), *F*. *vesca* (WGS_1: AEMH01010803; WGS_2: AEMH01014087), *P*. *persica* (AEKV01002084). TFs protein sequences from *ERF2* are: *C*. *papaya* (ABIM01003643), *A*. *thaliana* (BAC42579), *M*. *domestica*_*1* (ADE41128), *M*. *domestica*_*2* (ADE41114), *V*. *vinifera* ([1]: CBI28202; [2]: CBI36313), *S*. *lycopersicum* (AAL75809; WGS_1: AEKE02002405; WGS_2: AEKE02023116), *F*. *vesca* (AEMH01012502), *P*. *persica* (AEKW01000994).

Three closely-related *ERF*/*AP2* genes had diverse expression throughout papaya ripening, with an *AP2* gene (AT4G39780) and an *ERF* gene (AT1G15360) being up-regulated (Table 2). While some genes from *AP2*/*ERF* family are related to improving *Arabidopsis* resistance to abiotic stress [19], others have been shown to be responsible for controlling tomato ripening [20,21]. Specific *APETALA2* genes (so called *SlAP2a* to *SlAP2e* genes) are expressed throughout normal tomato ripening and *SlAP2a* gene may balance the activities of positive ripening regulators as a negative feedback loop. Moreover, a previous study showed an *APETALA2* gene subclade IIIc (*COLD BIND FACTOR II* gene) is involved in regulation of apple pulp softening during cold storage and/or ethylene treatment [22]. In fact, papaya *ERF2* and *RAP2* putative proteins share higher similarity to diverse *APETALA2* proteins from apples and tomato (Figure 6) rather than the *SlAP2a* protein from tomato.

Three genes members of auxin signalling pathway (*ARF18*, *AXR1* and *ARP*), a hormone that impairs ripening [6], were identified in our experiments. Whereas *ARP* was the only up regulated gene during ripening, *ARF18* was phylogenetically close to *SlARF* family, the auxin response factors from *S*. *lycopersicum* (Figure 5). These data suggest that ripening in papaya involves auxin signalling, in common with other fruits [23,24].

Many different TFs are involved in control of ripening in fleshy fruits. In tomato, master regulators include *MADS*-box (*SEP4like*, *RIN*, *TDR4*, *TAG1*, *TAGL1*), *SBP*-box (*CNR*), *HBbox* (*LeHB1*) and *NAC* genes [25]. Strawberry ripening involves a *SEP1*/*2like* gene (*FaMADS9*) [26], and for banana a *SEP3like* gene (*MaMADS2*) [27]. In bilberry, accumulation of anthocyanins is controlled by an *SQUAMOSAclass MADS*-box TF *VmTDR4*, orthologous to the *TDR4* gene in tomato [28]. Papaya shows range of TFs which are homologous to these master regulators and show ripening-related changes in gene expression (Table 2). Despite each species seeming to have specific sets of ripening-related transcription factors, their exact functions in papaya fruits remain to be elucidated.

#### Cell wall-related genes

Papaya pulp softening shows a remarkable change during ripening, and this is thought to be due to the activities of cell wall hydrolases [29]. Seven putative cell wall-related genes were differentially expressed: four related to pectin hydrolysis (α-galactosidase - AT3G26380; pectate lyase - AT4G24780; pectin methylesterases - AT5G48450 and AT4G12420), two related to cellulose hydrolysis and rearrangement (cellulase - AT4G28320; xyloglucan endotransglycosylase - AT2G06850) and one related to cell expansion (expansin - AT2G39700). Mostly genes related to PGs and expansin were up-regulated while those related to PMEs, PLs and glucosidases were down-regulated.

**Figure 7:**
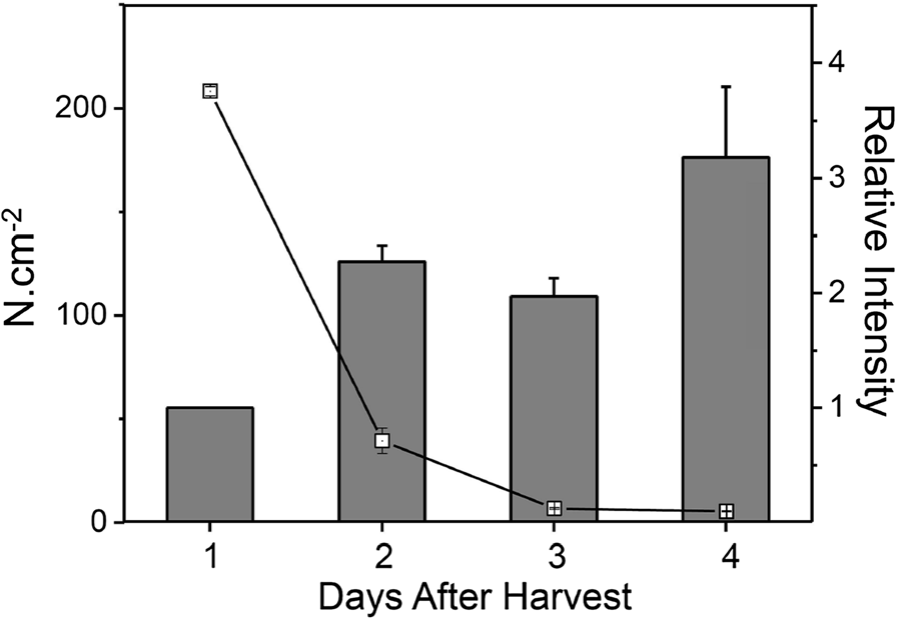
**Correlation between pulp softening and expansin gene expression **(***EXP - *AT2G39700**) **during papaya ripening. **Pulp firmness (Open squares) was monitored through the ripening process by a texturometer (Fabi et al., 2007) and measures are given by N.cm^-2^. The mRNA abundance of expansin gene is indicated by column height. Expression values in unripe fruit at the first day after harvest were set to 1. Error bars indicate SDs of the mean (*n*=12 for papaya texture analysis and *n*=4 for qPCR analysis).

The up-regulation of an α-galactosidase gene (*AGAL*) observed in our experiments is consistent with previous data reported by Soh et al. [30] and Nogueira et al. [31] on ripening of papaya fruit. On the other hand, changes in pectate lyases (*PL*) were detected, but the reduced levels contrast with the expected role of the enzyme during ripening. In other fruits, such as bananas [32] and mangoes [33]*PL* is thought to contribute to the pectin disassembly that leads to the pulp softening of ripe fruits. Pectin methylesterases were also apparently down-regulated during ripening. Solubilisation of pectin in ripe papayas may be governed principally by the action of polygalacturonases on previously de-esterified pectin chains [29,34] in addition to the action of α-galactosidases. Cellulose and hemicellulose processing in the papaya pulp could not be inferred from our results, since the genes related to hydrolysis and rearrangements of cellulose (cellulase and xyloglucan endotransglycosylase, respectively) were down-regulated during papaya ripening, in contrast to the up-regulation in ripe tomato and strawberry [35,36]; respectively). Gaete-Eastman et al. [37] observed expansin gene expression was up-regulated during ripening in mountain papaya and it was inversely correlated to pulp texture. This pattern of expression is in agreement with the expansin gene from ‘Golden’ papaya (Figure 7). Papaya EXP is related to the gene products encoded by *FaEXP4*, *PcEXP5* and *PpEXP2* from strawberry, pear and peach, respectively [38-40]; Figure 6). All of these genes are up-regulated during fruit ripening, suggesting conservation of gene action in the softening of a range of fleshy fruits species. However in some instances cell wall changes in papaya resembled events in non-fruit tissues. An *Arabidopsis* microarray study [14] investigating gene expression during 5 or 11-day-old hypocotyls (ratio 11-day/5-day) showed a transcription profile, at least for some of the cell wall-related genes, that was highly similar to that found in ripening papaya (Table 4). This reveals interesting characteristics of cell wall remodelling which may have evolved from the same ancestral Brassicales.

#### Response to stress and plant defence

No representative differences in expression of probe-sets related to ‘response to stress’ (six probe-sets up-regulated and six probe-sets down-regulated) were observed, and only two out of six ‘plant defence response’ probe-sets were up-regulated. However, genes from other GO classes may be considered based on their putative functions. Acyl-CoA oxidases (*ACXs*) are members of ‘lipid metabolism’ GO, and have a key role in the jasmonic acid (JA) biosynthesis, an important compound in protection against pathogens and insects [41]. The up-regulated papaya *ACX* gene could contribute to the defence system during ripening, since it showed a 5-fold increase in transcript abundance. Papaya ACX protein shared high similarity with that from tomato, where a peroxisomal ACX protein is critical for the β-oxidation through JA biosynthesis and systemic wound signalling, indicating this gene might also be responsible for JA biosynthesis in papaya [42]. Two putative papaya *ERD3* and *ERD4* genes (AT2G39750 and AT4G04340, respectively) were down-regulated. These proteins are methyltransferases that respond positively to abiotic stresses such as cold treatment and prolonged mild osmotic stress [43].

Analysis of the papaya transcriptome reveals that like tomato there is overexpression of heat-shock protein (*HSPs*) genes. Heat-shock proteins can work as chaperones in protein folding; under stress conditions they can re-establish normal protein conformation [44] and degrade damaged or misfolded peptides [45]. Genes normally associated with response to abiotic stress are often seen expressed in ripening fruits [8,46].

Chitinases and thaumatins were over-expressed in grapes, conferring plant protection against saprophytic organisms [47], but down-regulated in tomato instead [15], none of these genes were differentially expressed in papaya. Only some genes related to protein inhibition and those of so-called leucine repeat proteins (LRRs), which are very important in defence against pathogenic fungi [48] were induced. Regarding redox regulation, ascorbic acid (Vitamin C) accumulates in these three fruits [49-51], but only papaya cross-species microarray revealed genes related to ascorbic acid biosynthesis: the up regulated monodehydroascorbate reductase and an L-galactose dehydrogenase genes.

## Conclusions

The heterologous hybridization microarray was successfully applied in the study of transcripts changes associated to papaya fruit, a commercially relevant crop from a non-model organism. The ripening of papaya represents a time-course of cellular metabolism changes, characterized by differential expression of numerous genes involved in primary metabolism, hormonal signalling, transcriptional regulation, abiotic stress and cell wall metabolism. Among the genes identified are transcription factors (TFs) of the *MADS*-box, *NAC* and *AP2*/*ERF* gene families, which are master regulators in other fruits indicating conservation of function for ripening control genes across different taxa. Moreover, data revealed cell wall-related gene expression was more similar to *Arabidopsis* hypocotyl development profiles than those from other fleshy fruit perhaps revealing characteristics of cell wall remodelling mechanisms specific to the Brassicales.

The phylogenetic relationship between transcription factors of fleshy fruits might indicate shared transcriptional regulatory networks. Although data presented in the manuscript is not enough to indicate how climacteric and non-climacteric fruits evolved, and some genes specific to papaya or those specifically expressed in fleshy organs might have been overlooked with the heterologous microarray, other approaches, e.g. RNAseq, are likely to contribute to new perspectives on how different fleshy fruits respond to ripening.

## Methods

### Samples

Papaya fruit (*Carica papaya* L. cv. ‘Golden’) were harvested at colour break to ¼ yellow (around 150 days after anthesis). One replicate of each unripe and ripe fruit samples for microarray assay was obtained from a previous study in 2007 [3]. The other two replicates of unripe and ripe samples for microarray and also for quantitative PCR analyses were obtained in 2010. Soon after harvest, the initial respiration and ethylene levels were determined [3]. Each experimental sample (unripe papaya and successive time points during the ripening process) comprised 12 individual fruits. After removal of the peel and seeds, the sliced pulp of each fruit was frozen in liquid N_2_ and stored at −80°C.

### Genomic DNA and total RNA extractions

Genomic DNA and total RNA extraction protocols were the same as previously described [29]. Total RNA was purified using the “RNeasy® Kit” (Qiagen). Nucleic acids were quantified in the NanoDrop® ND-1000 (Nanodrop Technologies^©^) and gel analyzed to verify their integrity [52]. Total RNA were also analyzed with Agilent 2100 Bioanalyser” (Agilent Technologies^©^) to confirm its integrity before each hybridization.

### Genomic DNA hybridization, probe-selection and cRNA hybridization

Genomic DNA was labelled with the Bioprime DNA labelling System kit (Invitrogen™), hybridized with Affymetrix GeneChip ATH1-121501 (Affymetrix) for 16 hours at 45°C using standard hybridization protocols (Affymetrix^©^) and analyzed by computed scanning. Using a *perl* script [53] and a range of user-defined threshold values (from 0 to 500), chip definition files (CDF) were created with papaya genomic DNA hybridization data (mask files). Probe-pairs were retained for analysis if their signal values were greater than the defined threshold. Probe-sets were retained if they contained more than one probe-pair. Total RNA (5 μg) from papaya pulp was reverse-transcribed to generate first strand cDNA containing a 5’-T7 RNA polymerase promoter sequence. Double stranded cDNA was synthesized using standard protocol, and the resulting samples were *invitro* transcribed by T7 DNA polymerase using biotinylated nucleotides to generate complementary RNAs (cRNAs). Purified cRNA (15 μg) were heat-fragmented and hybridized to ATH1-121501 for 16 hours at 45°C. The complete protocol has already been published [11]. For each fruit stage (unripe and ripe), a triplicate of hybridization was undertaken and all the hybridizations have been submitted to GEO (http://www.ncbi.nlm.nih.gov/geo; accession number GSE38105).

### Data analyses

Microarray Analysis Suite (MAS Version 5.0; Affymetrix) was used to generate .CEL files for each RNA hybridization. These files were loaded into GeneSpring version 7.2 (Agilent Technologies) software using the Robust Multichip Average (RMA) pre-normalization algorithm [54]. The computer files that were being loaded in GeneSpring were filtered using the CDF files generated using the genomic DNA hybridization. For each replicate array, each probe-set signal value from ripe samples was compared to the probe-set signal value of unripe samples to give gene expression ratios. Differentially expressed genes were identified using one-way ANOVA with a Benjamini and Hochberg false discovery rate multiple testing correction. The differentially expressed genes had their log_2_ signal intensities computed from the replicate chips and hierarchical clustering was carried out using the EPCLUST software [55] with a complete linkage algorithm and the Euclidian distance on normalized vectors of length 1 (chord distance) as parameters.

### Comparative analyses using PageMan and MapMan

Normalized gene expression data were subjected to analysis of functional categories using PageMan and MapMan functional categories [56,57]. Using the Wilcoxon test it was possible to assume whether significantly more genes in ripe vs. unripe point were up-regulated when normalized to their average expression. Expression data (log_2_ fold) from papaya microarray (ripe X unripe) was loaded and analysed in MapMan software for visualization of the cellular pathway [13] against *Arabidopsis* mapping (Ath_AFFY_ATH1_TAIR8_Jan2010). In the same way, expression data (log_2_ fold) from *Solanum lycopersicum* cultivar MicroTom (ripe X mature green; [58]) and *Vitis vinifera* cv. Pinot Noir (post-veráison X pre-véraison; [59]) were loaded and analysed in MapMan software, using data from Ozaki et al. [15] against *S*. *lycopersicum* mapping (Slyc_AFFY_SGN_BUILD2_070709) and Pilati et al. [14] against *V*. *vinifera* mapping (Vvin_AFFY_09), respectively. Log_2_ fold values of differentially expressed probe-sets from three species were compared and a Venn diagram enclosing only the log_2_ values, not the probe-set IDs (since three different platforms were used) was created. For papaya up-regulated probe-sets, the *Arabidopsis* gene annotations and functional classifications were analysed using the gene ontology (GO) function of the GeneSpring software.

### Quantitative analysis of gene expression by real time-PCR (*qPCR*)

For the validation of putative papaya genes identified by the XSpecies hybridization, the *Arabidopsis* genes represented in the hybridized chips were aligned individually against the WGS database using the *BLASTN* tool (score ≥ 100 and e-value ≤ 1e^-30^ as cut-off values). Papaya putative coding sequences that were satisfactory aligned to *Arabidopsis* genes were evaluated following the ‘Minimum Information for Publication of Quantitative Real-Time PCR Experiments – MIQE’ [60] and also according to the parameters: (1) pair of primers with melting temperature of 60°C and absence of primers-dimers and hairpins; (2) amplicon size between 75 and 200 bp; (3) amplicon evaluation with the mfold program [61]; and (4) amplicon alignment with an unique papaya WGS sequence (in order to not amplify two closely-related genes or a duplicated one). Twenty-one putative genes satisfied the above mentioned criteria and had their expression levels quantified. Primers were designed using Primer 3 (v.0.4.0) tool [62], and the sequences are shown in Additional file 9. As an internal controls, the putative actin gene located on chromosome LG9 contig 1059 (GenBank accession no. **ABIM01001059**) was used with sense (5^′^-CGT GAC CTT ACT GAT CAC TTG-3’) and reverse (5’-GTC AAG GGC AAT GTA AGA CAG-3’) primers in combination with the 18S rRNA (GenBank accession no. **U42514**) with sense (5^′^-AAA CGG CTA CCA CAT CCA AG-3’) reverse (5’-CGA AGA GCC CGG TAT TGT TAG GG-3’) primers. After on-column digestion of DNA with DNase RNase-free (NucleoSpin® - Macherey Nagel^©^), first-strand cDNA was synthesized with random primers from 1 μg total RNA using the ImProm-II Reverse Transcription System (Promega). For primer testing and identity confirmation, the fragments from preliminary PCR were cloned and sequenced. Real-time PCR was performed using the Platinum SYBR Green qPCR Supermix UDG (Invitrogen) in a “Rotor-Gene 3000 four channel Multiplexing System” (Corbett Research). The amplification was carried out under the following conditions; 50°C for 2 min followed by an initial denaturation step at 95°C for 2 min, 40 cycles at 95°C for 15 s, 60°C for 30 s, and 72°C for 30 s. Non Template Controls (NTCs) and melting curve analyses of amplicons were analysed for all experiments. The threshold cycle (Ct) values of the four technical replicate reactions were averaged using the Rotor-Gene 3000 software and quantification was performed using the relative standard curve method [63]. Samples used in *qPCR* experiments were a mixture of, at least, 32 fruits from 2010 second and third biological replicates [8] (Additional file 5). Developmental parameters included ripening-related events such as CO_2_ and ethylene production as well as pulp softening [3]. The results of the standard curves calculation are in Additional file 10. Data were analysed against the first day after harvest by one-way ANOVA, and means were compared using the Tukey test at α<0.05. Statistical analysis was carried out using OriginPro version 8 (OriginLab®).

### Comparative biology analysis between fleshy fruit organisms

In order to identify genes similarities shared between some fleshy fruit organs, a comparative biology analysis was done using qPCR tested papaya genes. The validated probe-sets were aligned to WGS database of papaya genome and the putative corresponding proteins were identified. Sequential TBlastN analyses were done using the putative papaya proteins and the WGS database for fleshy fruit organisms such as tomato (*S*. *lycopersicum*), strawberry (*F*. *vesca*), peach (*P*. *persica*) and grape (*V*. *vinifera*), and the corresponding proteins were also obtained for each organism. If otherwise stated, all sequences were identified from this manner. Together with *Arabidopsis* proteins and some other proteins, they were aligned using ClustalW [64] and phylogenetic trees were drawn using the Phylodendron^©^ software [65]. The Genbank ID and sequences of proteins used in comparative biology analysis are in Additional file 11.

## Abbreviations

Blast: Basic Local Alignment Search Tool; GO: Gene Ontology; qPCR: quantitative PCR; PCR: Polymerase chain reaction; TF: Transcription factor; WGS: Whole genome shotgun; XSpecies Microarray: Cross Species Microarray.

## Competing interests

The authors declare that they have no competing interests.

## Authors’ contributions

JPF wrote the article and performed the research. GBS and JRON corrected the manuscript and helped in its preparation. NSG, MRB and STM helped in data analysis and manuscript preparation. FML and BRC helped in manuscript preparation. All authors read and approved the final manuscript.

## Supplementary Material

Additional file 1 **Effect of *Carica papaya *genomic DNA (gDNA) hybridization on probe-pairs and probe-sets from the ATH1-121501 chip. **This additional figure describes how different values of hybridization intensity threshold (masks) affect the probe-sets and probe-pairs retained after papaya genomic hybridization in *A*.*thaliana* commercial chip. Click here for file

Additional file 2 **Differentially expressed probe**-**sets identified in papaya fruit. **This table is a full version (edited in Excel program) of Table 1 with crude data (19,286 probe-sets) and treated data (414 probe-sets). It describes the principal characteristics of probe-sets retained after threshold cut-off and comparison between ripe X unripe fruit, that includes systematic and common names of probe sets, normalized and raw values, fold time changes and *p* values, as well as GO identities for biological process, molecular function and cellular component. Click here for file

Additional file 3 **Hierarchical clustering of ATH1-121501 probe-sets hybridized with papaya RNA. **This figure describes the hierarchical clustering of the 414 probe-sets identified by the XSpecies microarray technique, showing different clusters of gene expression. Click here for file

Additional file 4 **Differentially expressed probe-sets identified in papaya fruit. **This table is a shorter version of Additional file 2, also edited in Excel program. However this table shows the probe-sets collected for generation of GO plot figure by names (Figure 1A and B) and also describes the parameters of Genespring software from statistical analyses. Click here for file

Additional file 5 **Ripening analysis from three different samples of papaya fruit. **This table describes the main parameters that were used for classifying as unripe (green) and ripe (yellow) papayas. This includes respiration (CO_2_ production), ethylene production and pulp firmness. Click here for file

Additional file 6 **Differentially expressed probe-sets identified in papaya fruit. **This table shows, in a reduced form, the probe-sets that were differentially expressed according to fold changes between ripe X unripe, but with a statistical *p*-value cut-off of 0.10, which returned 1,091 differentially expressed probe-sets. Click here for file

Additional file 7 **Overview of cell function transcripts from tomatoes, grapes and papayas in response to ripening. **This figure describes the cell function transcripts from tomato, grape and papaya fruits when analysed with the MapMan software.These values (and identities) were the same used to structure the Venn diagram in Figure 2. Click here for file

Additional file 8 **Differentially expressed transcription factors from papaya, tomato and grape. **This table is a simplified version of Table 2 (edited in Excel program) and Figure 3. The table only returns the results from MapMan analysis of overview ripening regulation of papaya fruit, but there is no comparison of transcription factors between three species. Click here for file

Additional file 9 **Nucleotide sequences used in qPCR. **This table describes the primers used in Real Time-PCR analyses. Click here for file

Additional file 10 **Standard curve calculation of Real Time-PCR primers. **This table describes the efficiency values, as well the standard curves for all primers used in Real Time-PCR experiments. Click here for file

Additional file 11 **Genbank ID and sequences of proteins used for comparative biology analyses. **This is compacted (zipped) file which contains all sequences of proteins used for generating the phylogenetic trees in order to analyse the comparative biology between different. Click here for file
