# Supplementary material for: Analysis of ripening-related gene expression in papaya using an Arabidopsis-based microarray
Source: BMC Plant Biol. 2012 Dec 21;12:242. doi: 10.1186/1471-2229-12-242 (PMC3562526; doi:10.1186/1471-2229-12-242)
Supplement: Additional file 1 — Effect of Carica papaya genomic DNA (gDNA) hybridization on probe-pairs and probe-sets from the ATH1-121501 chip. This additional figure describes how different values of hybridization intensity threshold (masks) affect the probe-sets and probe-pairs retained after papaya genomic hybridization in A.thaliana commercial chip. [file 1471-2229-12-242-S1.pdf]

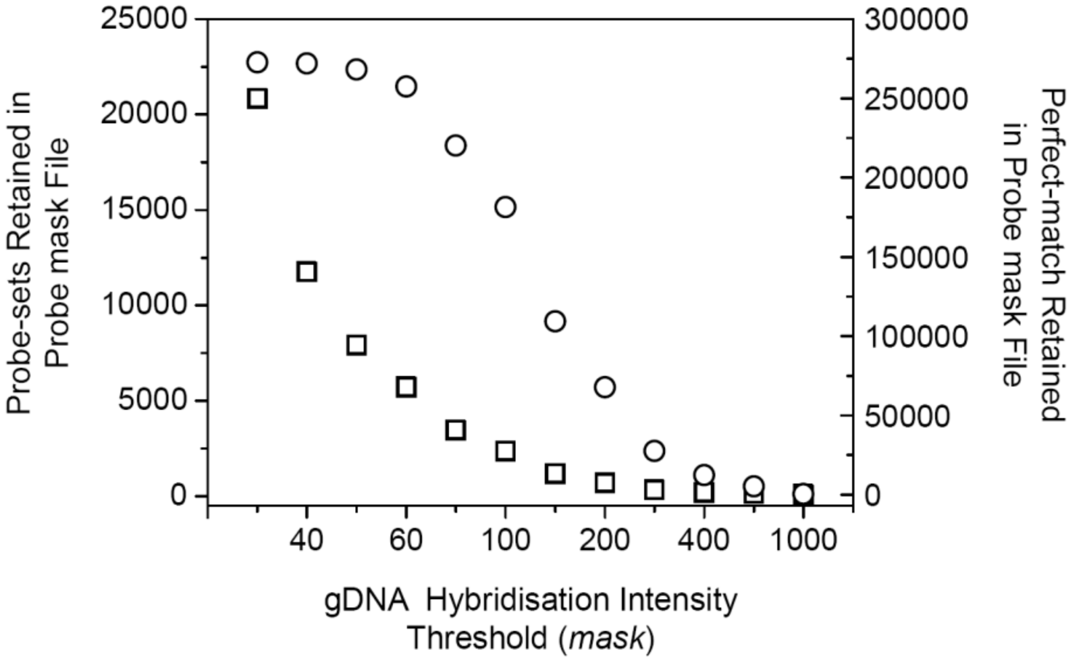

Supplementary Figure 1. Effect of *Carica papaya* genomic DNA (gDNA) hybridisation on probe-pairs and probe-sets from the ATH1-121501 chip. Different values of hybridisation intensity threshold (*masks*) affect both probe-sets and probe-pairs retained. Open circles are scaled to the left side of the graphic (i.e. probe-sets used in probe mask files) while open squares are scaled to the right side (i.e. probe-pairs used in probe mask files).
