# Supplementary material for: Analysis of ripening-related gene expression in papaya using an Arabidopsis-based microarray
Source: BMC Plant Biol. 2012 Dec 21;12:242. doi: 10.1186/1471-2229-12-242 (PMC3562526; doi:10.1186/1471-2229-12-242)
Supplement: Additional file 3 — Hierarchical clustering of ATH1-121501 probe-sets hybridized with papaya RNA. This figure describes the hierarchical clustering of the 414 probe-sets identified by the XSpecies microarray technique, showing different clusters of gene expression. [file 1471-2229-12-242-S3.docx]

**Additional File 3.** **Hierarchical clustering of ATH1-121501 probe-sets hybridised with papaya RNA.** The hierarchical clustering of 414 *A. thaliana* probe-sets identified by the XSpecies microarray technique was done using the EPCLUST software. Each row represents the identified probe-set with log_2_ normalized intensities shown over the different stages: U, unripe; R, ripe. Total red and total green reflect putative transcriptional activation (≥3-fold) and repression (≤3-fold) respectively. The dendrogram shows the relationships between clusters of genes with similar expression pattern. Clusters generated by the cut of the tree at 0.75 height (distance) are displayed in Roman numbers.
