# Supplementary material for: Analysis of ripening-related gene expression in papaya using an Arabidopsis-based microarray
Source: BMC Plant Biol. 2012 Dec 21;12:242. doi: 10.1186/1471-2229-12-242 (PMC3562526; doi:10.1186/1471-2229-12-242)
Supplement: Additional file 5 — Ripening analysis from three different samples of papaya fruit. This table describes the main parameters that were used for classifying as unripe (green) and ripe (yellow) papayas. This includes respiration (CO2 production), ethylene production and pulp firmness. [file 1471-2229-12-242-S5.pdf]

Additional File 5. Ripening analysis from three different samples of papaya fruit.

Parameters	First Sample		Second Sample		Third Sample	
	Unripe	Ripe	Unripe	Ripe	Unripe	Ripe
CO_2 (*mg.Kg*$^{-1}$*.h*$^{-1}$)	2.42 ± 0.44	60.04 ± 4.05	11.19 ± 1.33	55.18 ± 2.36	12.34 ± 3.15	57.74 ± 5.31
Ethylene (*mL.Kg*$^{-1}$*.h*$^{-1}$)	0.19 ± 0.03	4.08 ± 0.37	0.14 ± 0.07	8.36 ± 1.08	0.24 ± 0.31	7.91 ± 0.96
Pulp firmness (*N.cm*$^{-2}$)	12.49 ± 0.84†	0.34 ± 0.11	211.21 ± 8.54††	5.24 ± 0.78	208.79 ± 1.04††	4.30 ± 0.56

† Pulp firmness measured with a "TA-42 knife blade with chisel end" probe [3];
†† Pulp firmness measured with a "TA-53 point 3 mm diameter" probe [8].

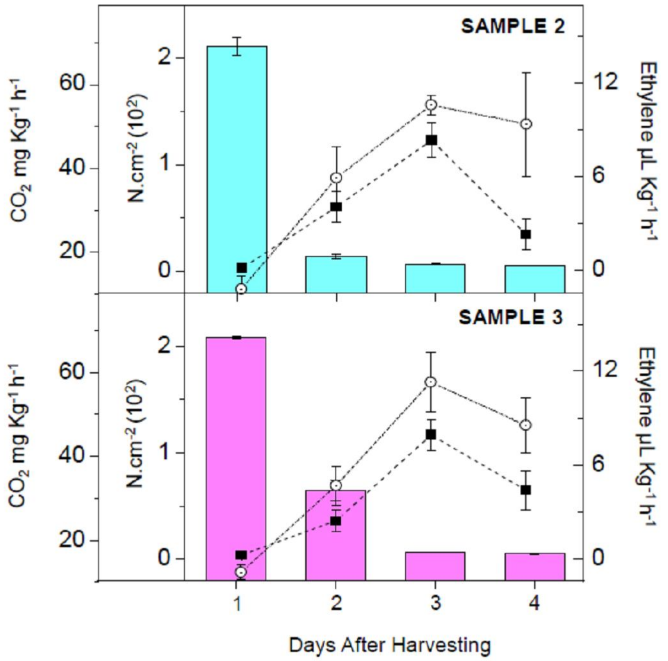

Additional File 5. Characterisation of papaya fruit ripening. Unripe papaya fruit had the amount of CO_2 produced by respiration (Open circles), production of endogenous ethylene (Black squares), and pulp firmness (Bars) monitored. Error bars indicate SDs of the mean (n=12) for each sampling (sample 2 and 3) [8]. Unripe and ripe fruit comprise data presented in the graphic for the 1st and 3rd days after harvesting.
