# Supplementary material for: Analysis of ripening-related gene expression in papaya using an Arabidopsis-based microarray
Source: BMC Plant Biol. 2012 Dec 21;12:242. doi: 10.1186/1471-2229-12-242 (PMC3562526; doi:10.1186/1471-2229-12-242)
Supplement: Additional file 7 — Overview of cell function transcripts from tomatoes, grapes and papayas in response to ripening. This figure describes the cell function transcripts from tomato, grape and papaya fruits when analysed with the MapMan software.These values (and identities) were the same used to structure the Venn diagram in Figure 2. [file 1471-2229-12-242-S7.pdf]

A

B

C

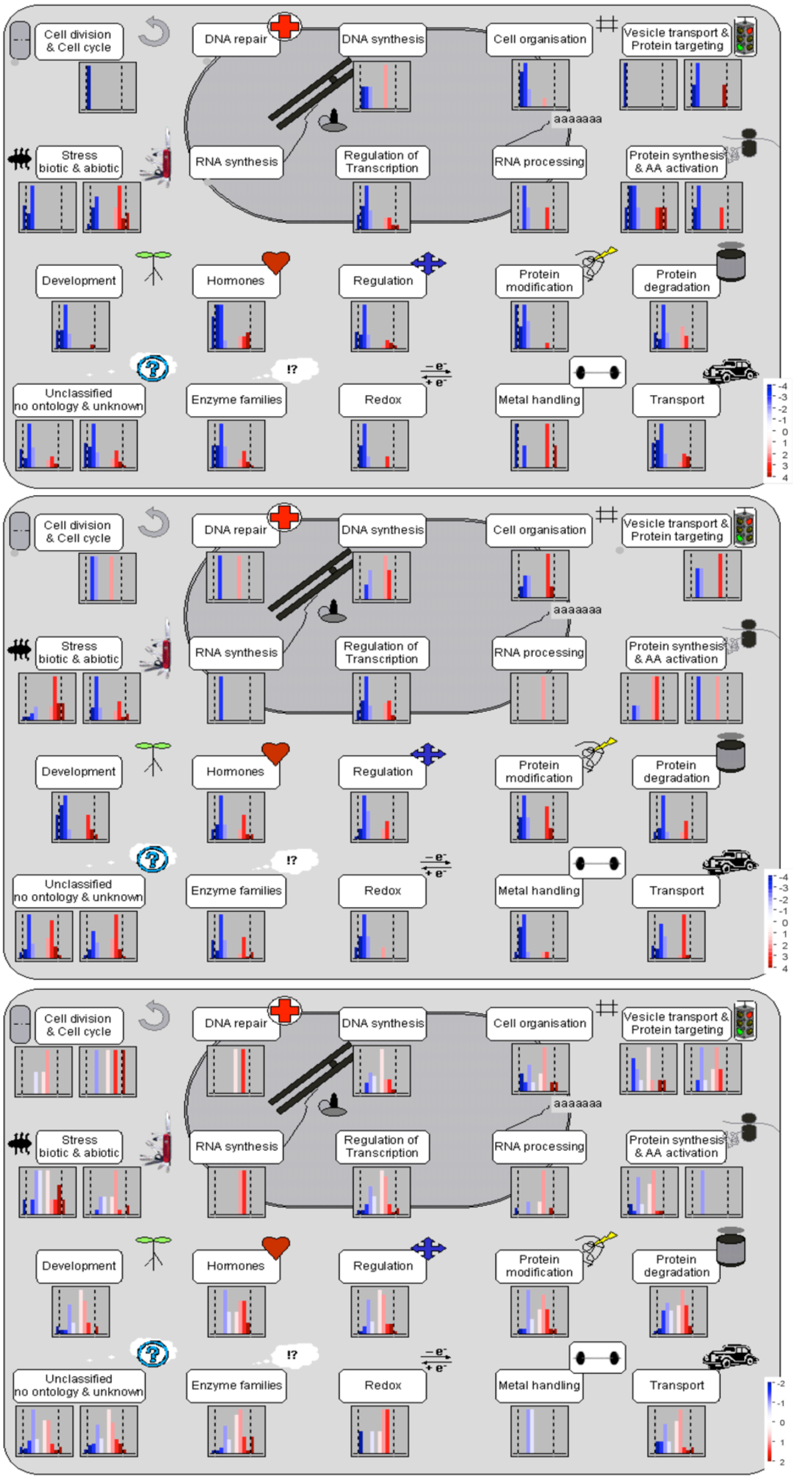

Supplementary Figure 3. Overview of cell function transcripts from tomatoes, grapes and papayas in response to ripening. Cell function transcripts from tomato (A), grape (B) and papaya (C) fruits were analysed using the MapMan software uploaded with microarrays results. Blue and red represent a decrease and an increase of expression respectively, relative to unripe fruits. It is visible 853 transcripts (from 1144) for tomato (> 2.00 fold), 992 transcripts (from 1150) for grape (> 2.00 fold) and 961 transcripts (from 1091) for papaya (> 1.25 fold). Tomato microarray data was downloaded from http://ted.bti.cornell.edu (Ozaki et

al., 2010); grape microarray data was downloaded from http://biomedcentral.com (Pilati et al., 2007).
