# Supplementary material for: Analysis of ripening-related gene expression in papaya using an Arabidopsis-based microarray
Source: BMC Plant Biol. 2012 Dec 21;12:242. doi: 10.1186/1471-2229-12-242 (PMC3562526; doi:10.1186/1471-2229-12-242)
Supplement: Additional file 9 — Nucleotide sequences used in qPCR. This table describes the primers used in Real Time-PCR analyses. [file 1471-2229-12-242-S9.docx]

| **Supplementary Table 1.** Nucleotide sequences used in qPCR. | | |
| --- | --- | --- |
| *Gene* | *Primer name* | *Sequence (5’→3’)* |
| ENOL | >enol_f | GTGCTCCCAGTCATCTTGGT |
|  | >enol_r | TGGTGAAGCTCTCAAGGATC |
| FRUCT | >fruct_f | GACTGATGGATCCGTAGTG |
|  | >fruct_r | ACGCTGAAGAAGGATGGAG |
| LIP3 | >lip3_f | TGAACAGATGCTGCCTGAAG |
|  | >lip3_r | CCTCCTAATGAATGCCCAGT |
| LIP | >lip_f | CTCGAATTCGTCAACTTCACC |
|  | >lip_r | GTTTGGGTTGCGGTAACATTG |
| MEV | >mev_f | AGCTGCAGTTGGGAAGTTG |
|  | >mev_r | GAAATCCGTAGTCGTGCTG |
| ERF2 | >erf2_f | ATCAGAATAGCTGCCTCGTC |
|  | >erf2_r | GAGAGTATGGCTTGGGACAT |
| RAP2.1 | >rap2.1_f | AGGATGAGGAAGTGGGGTA |
|  | >rap2.1_r | ATGGACCACGGAGGTAGAA |
| ARF18 | >arf18_f | GTGTCCTTGCTACTGCTTCT |
|  | >arf18_r | GCGCATGCCTACAGAAAATC |
| AXR1 | >axr1_f | GTGATTTGGGTTGCAATGGG |
|  | >axr1_r | GCAATTCCTCCAATGAAGGC |
| ARP | >arp_f | AGGGAGGTCTGCCATTACAT |
|  | >arp_r | CGAGTCATGAGTTGGCAAGA |
| HSP70 | >hsp70_f | AGCTAGCCTTGCCATATCC |
|  | >hsp70_r | CTGCAATCCGATCATAGCG |
| ACX | >acx_f | CTTCGCCAACAATTTGGTCC |
|  | >acx_r | AGGGCATGAACATCTCCAAC |
| ERD3 | >erd3_f | CCGGATATTGAGACCTGGT |
|  | >erd3_r | CCCTCAGATGTGTCACGTA |
| ERD4 | >erd4_f | AAGAGCGATCAGAAGCGGTA |
|  | >erd4_r | CCGTGTCATTGGTGCATTGA |
| A-GAL | >a-gal_f | GTATAGGCGGAAGGTGGAT |
|  | >a-gal_r | AAGGCCACCTCTCTGGAT |
| EXP | >exp_f | GTAGCCCGTCAATCTTCGT |
|  | >exp_r | GGCCAAGTCGAAGTGAGAA |
| PL | >pl_f | CATGTTCTTGTCCTGCGTGT |
|  | >pl_r | CCATCCACGGCTCTAATTTC |
| PME1 | >pme1_f | GTGGTTTGTTCCTCAGCACA |
|  | >pme1_r | TGGAACGTAACTGCAAGTGG |
| PME2 | >pme2_f | GCAAGCTTTAGGGGTGTTGA |
|  | >pme2_r | AGGCCTGCAGAGCTTATTGA |
| CELL | >cell_f | GCCTCCAGACCCATTTCTTT |
|  | >cell_r | CATCGAAGATGGTGACAACG |
| XTH | >xth_f | GCACTCAGGAGGAAGAGTA |
|  | >wth_r | GTGGGACCAGAAGGAGTTT |
