# Supplementary material for: Analysis of ripening-related gene expression in papaya using an Arabidopsis-based microarray
Source: BMC Plant Biol. 2012 Dec 21;12:242. doi: 10.1186/1471-2229-12-242 (PMC3562526; doi:10.1186/1471-2229-12-242)
Supplement: Additional file 10 — Standard curve calculation of Real Time-PCR primers. This table describes the efficiency values, as well the standard curves for all primers used in Real Time-PCR experiments. [file 1471-2229-12-242-S10.docx]

| **Supplementary Table 2.** Standard curve calculation of Real Time-PCR primers. | | | |
| --- | --- | --- | --- |
| *Name of the gene* | *Efficiency*  *(10^-slope^)* | *y=ax+b* | *R^2^* |
| ENOL | 2.02 | -3.2695x + 18.709 | 0.9909 |
| FRUCT | 2.31 | -2.7458x + 31.126 | 0.9867 |
| LIP3 | 2.02 | -3.2647x + 24.213 | 0.9994 |
| LIP | 1.81 | -3.8693x + 26.473 | 0.9972 |
| MEV | 1.94 | -3.4742x + 23.389 | 0.9998 |
| ERF2 | 1.82 | -3.8546x + 35.543 | 0.9922 |
| RAP2.1 | 2.03 | -3.2558x + 22.585 | 0.9949 |
| ARF18 | 2.00 | -3.3269x + 23.503 | 0.9963 |
| AXR1 | 1.81 | -3.8919x + 29.025 | 0.9464 |
| ARP | 1.89 | -3.6026x + 21.797 | 0.9898 |
| HSP70 | 1.98 | -3.3746x + 23.362 | 0.9998 |
| ACX | 1.97 | -3.3908x + 20.930 | 0.9983 |
| ERD3 | 2.10 | -3.0939x + 21.859 | 0.9999 |
| ERD4 | 1.98 | -3.3708x + 21.343 | 0.9969 |
| A-GAL | 2.20 | -2.9239x + 25.249 | 0.9872 |
| EXP | 2.00 | -3.3321x + 27.523 | 0.9885 |
| PL | 1.98 | -3.3722x + 23.508 | 0.9954 |
| PME1 | 1.94 | -3.4841x + 27.584 | 0.9906 |
| PME2 | 1.93 | -3.4997x + 28.439 | 0.9999 |
| CELL | 1.86 | -3.7239x + 24.695 | 0.9942 |
| XTH | 1.74 | -4.1503x + 28.827 | 0.9898 |
| ACTIN | 1.82 | -3.8506x + 22.049 | 0.9967 |
| 18S | 2.16 | -2.9901x + 25.783 | 0.9688 |
