# Supplementary material for: Analysis of ripening-related gene expression in papaya using an Arabidopsis-based microarray
Source: BMC Plant Biol. 2012 Dec 21;12:242. doi: 10.1186/1471-2229-12-242 (PMC3562526; doi:10.1186/1471-2229-12-242)
Supplement: Additional file 11 — Genbank ID and sequences of proteins used for comparative biology analyses. This is compacted (zipped) file which contains all sequences of proteins used for generating the phylogenetic trees in order to analyse the comparative biology between different. [file 1471-2229-12-242-S11.zip › Fructo.rtf]

>A.thaliana (AAM13911)
MTSSNGDNKGLVVSFGEMLIDFVPTESGVSLSESSGFLKAPGGAPANVAIAVSRLGGRAAFVGKLGDDEFGHMLAGILRKNDVDDQGINFDKGARTALAFVTLRSDGEREFMFYRNPSADMLLRPDELNLELIRSAKVFHYGSISLITEPCRSAHMKAMEVAKEAGALLSYDPNLREPLWPSPEEARKQIMSIWDKADIIKVSDVELEFLTGNKTIDDETAMSLWHPNLKLLLVTLGENGCRYYTKDFHGSVETFHVDAVDTTGAGDSFVGALLNQIVDDQSVLEEEERLRKVLRFANACGAITTTKKGAIPALPTDCEALSFLKIQVE

>C.papaya (ABIM01006358)
MEEKMGRKSPLIVSFGEMLIDFVPDKAGLSLADSTGFLKAPGGAPANVACAISKLQGNSAFIGKVGDDEFGHMLVDILKKNGVKTEGVCFDPEARTALAFVTLKKDGEREFMFYRNPSADMLLKESELNMNMIKQAKIFHYGSISLISEPCRSAHLAAMKAAKSAGVMLSYDPNVRLPLWPSPQAAKDGIMSIWKEADFIKVSDDEVQFLTDKDSDKEDVVLSLWHDKLKLLVVTDGSKGCRYFTKVFKGRVAGFSVKPVDTTGAGDAFVGALLVSVANDPDEVKLREALSFANACGAISTTQKGAIPALPTISDALQLI

>V.vinifera_1 (CBI16962)
MLIDFVPDSAGVSLAESTGFLKAPGGAPANVACAITKLGGNSAFIGKVGDDEFGHMLVDILKKNGVNSEGVCFDAHARTALAFVTLKKNGEREFMFYRNPSADMLLTESELNMGLIKQAKIFHYGSISLISEPCRSAHMAAMKAAKEAGILLSYDPNVRLPLWPSAQAAIDGIKSIWNHADFIKVSDDEVGFLTQGDAEKEDVVLSLWHDNLKLLVVTDGEKGCRYFTKGFKGRVEGFSVQTVDTTGAGDAFVGALLVSIAQDPSIFQDEGKLKEALKYANACGAICTTQKGAIPALPTNSDALDLVNKSKAK

>S.habroichates (Q7XJ81)
MAVNGASSSGLIVSFGEMLIDFVPTVSGVSLAEAPGFLKAPGGAPANVAIAVTRLGGRSAFVGKLGDDEFGHMLAGILKTNGVQADGINFDKGARTALAFVTLRADGEREFMFYRNPSADMLLTPAELNLDLIRSAKVFHYGSISLIVEPCRAAHMKAMEVAKEAGALLSYDPNLRLPLWPSAEEAKKQIKSIWDSADVIKVSDVELEFLTGSNKIDDESAMSLWHPNLKLLLVTLGEKGCNYYTKKFHGTVGGFHVKTVDTTGAGDSFVGALLTKIVDDQTILADEARLKEVLRFSCACGAITTTKKGAIPALPTASEALTLLKGGA

>S.lycopersicum (Q42896)
MAVNGASSSGLIVSFGEMLIDFVPTVSGVSLAEAPGFLKAPGGAPANVAIAVTRLGGKSAFVGKLGDDEFGHMLAGILKTNGVQAEGINFDKGARTALAFVTLRADGEREFMFYRNPSADMLLTPAELNLDLIRSAKVFHYGSISLIVEPCRAAHMKAMEVAKEAGALLSYDPNLRLPLWPSAEEAKKQIKSIWDSADVIKVSDVELEFLTGSNKIDDESAMSLWHPNLKLLLVTLGEKGCNYYTKKFHGTVGGFHVKTVDTTGAGDSFVGALLTKIVDDQTILEDEARLKEVLRFSCACGAITTTKKGAIPALPTASEALTLLKGGA

>V.vinifera_2 (XP_002263733)
MTITSKSPLVVAFGEMLIDFVPDSAGVSLAESTGFLKAPGGAPANVACAITKLGGNSAFIGKVGDDEFGHMLVDILKKNGVNSEGVCFDAHARTALAFVTLKKNGEREFMFYRNPSADMLLTESELNMGLIKQAKIFHYGSISLISEPCRSAHMAAMKAAKEAGILLSYDPNVRLPLWPSAQAAIDGIKSIWNHADFIKVSDDEVGFLTQGDAEKEDVVLSLWHDNLKLLVVTDGEKGCRYFTKGFKGRVEGFSVQTVDTTGAGDAFVGALLVSIAQDPSIFQDEGKLKEALKYANACGAICTTQKGAIPALPTNSDALDLVNKSKAK

>F.vesca_1 (AEMH01014352)
MANDKGLIVSFGEMLIDFVPTVSGVSLAEAPGFLNAPGGAPANVAIAVSRLGGNASFVGKLGDDEFGHMLAGILKENNVSGEGIMFDQGARTALAFVTLRADGEREFMFYRNPSADMLLMPEELNLELIKSASVFHYGSISLIVEPCRSAHLKAMEVAKEAGALLSYDPNLRLPLWPSAEEAREQIMSIWDKAEVIKVSDVELEFLTGNPNIDDESALSLWHSNLKLLLVTLGVKAFRVTAVDTTGAGDSFVGALLAKIVDDQSVLENEQRLREVLKFANACGAITTTKKGAIPALPNESEVLALI

>F.vesca_2 (AEMH01014958)
LIVAFGEMLIDFVPDTAGVSLAESTGFLKAPGGAPANVACAITKLGGKSAFVGKVVGDDEFGHMLINILKTNGVNTEGVCVDTHARTALAFVTLRKDGEREFMFYRNPSADMLLKDSELNMDLIKKAKIFHYGSISLISEPCKSAHMAAMKAAKDAGILLSYDPNVRLPLWPSADAARDGIKSIWNQADFIKVVSDDEVQFLTQGDPNKEEVVLSLWHDNLKLLIVTDGEKGCRYFTKVFKGNVTGFAVKTVDTTGAGDAFVGSFLLSMAKDLSFANACGAICTTQKGAIPALPAESDALELI

>P.persica (AEJG01002103)
ESPLIVAFGEMLIDFVPDTAGVSLAESTGFLKAPGGAPANVACAITKLGGKSAFVGKVGDDEFGHMLINILKKNGVNAEGVCVDTHARTALAFVTLRKDGEREFMFYRNPSADMLLKDSELNMPLIKQAKIFHYGSISLISEPCRSAHMAAMKAAKDAGIMLSYDPNVRLPLWPSADAAREGIKSIWNQADFIKVVSDDEVQFLTQGDAEKDDVVLSLWHDNLKLLVVTDGEKGCRYYTKVFKGKVTGFSVKAVDTTGAGDAFVGSFLLSMAKDTKLKESLSFSNACGAICTTQRGAVPALPTQFAALELI

>S.lycopersicum_WGS1 (AEKE02005933)
IVCFGEMLIDFVPDSSGVSLAESTGFLKAPGGAPANVACAITKLEGTSAFIGKVGDDEFGRMLVDILKSNGVNSEGVLFDKHARTALAFVTLKKNGEREFMFYRNPSADMLLKDSELNLGLIKQAKIFHYGSISLITEPVRSAHMVAMKSAKDAGVLLSYDPNVRLPLWPSPEAAREGIKSIWNEADFIKVSDDEVNFLTQKDADKEETIMSLWHDRLKLLVVTDGEKGCRYFTKVFKGKVSGFSVKTVDTTGAGDAFVGSLLVSIAKDPDEEKLKKALKFSNACGAISTTQKGAIPALPSTADAQGLI
